# Geschlechtsunterschiede bei Aggression im Rahmen psychischer Störungen: Eine narrative Literaturübersicht

**DOI:** 10.1007/s00115-025-01934-3

**Published:** 2025-12-23

**Authors:** Manuel Krebs, Katja Bertsch, Sabine C. Herpertz, Ute Habel

**Affiliations:** 1 Klinik für Psychiatrie, Psychotherapie und Psychosomatik, Uniklinik RWTH Aachen, Aachen, Deutschland; 2 Lehrstuhl für Psychologie I Klinische Psychologie und Psychotherapie, Julius-Maximilians-Universität Würzburg, Würzburg, Deutschland; 3 Klinik für Allgemeine Psychiatrie, Universitätsklinikum Heidelberg, Heidelberg, Deutschland; 4 JARA-Institut INM-10, Forschungszentrum Jülich, Jülich, Deutschland

**Keywords:** Substanzabhängigkeitsstörungen, Psychotische Störungen, Borderline-Persönlichkeitsstörung, Einflussfaktoren, Biopsychosoziales Modell, Substance use disorders, Psychotic disorders, Borderline personality disorder, Influencing factors, Biopsychosocial model

## Abstract

Aggressives Verhalten weist markante Geschlechtsunterschiede auf. Kulturübergreifende Forschungsergebnisse zeigen, dass Männer häufiger körperliche Aggression ausüben, während Frauen tendenziell zu indirekten Formen aggressiven Verhaltens neigen. Aggression ist ein multifaktorielles Phänomen, das durch situative, genetische, psychologische und weitere Faktoren beeinflusst wird. Gleichzeitig ist sie als transdiagnostisches Merkmal in zahlreichen psychischen Störungen relevant, wobei geschlechtsspezifische Unterschiede und zugrunde liegende Einflussfaktoren bislang unzureichend untersucht sind. Viele Befunde stammen aus älteren Studien, und methodisch hochwertige, insbesondere verhaltensbasierte Untersuchungen liegen nur vereinzelt vor. Der vorliegende Beitrag gibt in Form einer narrativen Literaturübersicht einen Überblick über den aktuellen Forschungsstand zu Geschlechtsunterschieden bei Aggression bei psychischen Störungen. Exemplarisch werden Substanzabhängigkeitsstörungen, psychotische Störungen und Borderline-Persönlichkeitsstörung betrachtet. Implikationen für zukünftige Forschung sowie die klinische Praxis werden diskutiert.

## Abstract (en)

Aggressive behavior shows striking gender differences. Cross-cultural research shows that men are more likely to engage in physical aggression, while women tend to use indirect forms of aggressive behavior. Aggression is a multifactorial phenomenon influenced by situational, genetic, psychological and other factors. Despite being a transdiagnostic feature in numerous mental disorders, gender-specific differences and the underlying influencing factors have not yet been sufficiently investigated. Many findings originate from older publications and methodologically sound and in particular behavior-based investigations are rare. This article provides a narrative literature review that summarizes the current state of research on gender differences in aggression in selected mental disorders, including substance use disorders, psychotic disorders and borderline personality disorder. The implications for future research and the clinical practice are discussed.

## Geschlechtsunterschiede bei Aggression

Aggression ist ein tief verankertes menschliches Verhalten, das in verschiedenen sozialen Kontexten auftritt. Aus evolutionärer Perspektive erfüllt aggressives Verhalten adaptive, überlebenswichtige Funktionen. In modernen Gesellschaften überwiegt eine negative Konnotation und aggressives Verhalten wird meist gesellschaftlich abgelehnt und sanktioniert. Seit jeher werden geschlechtsspezifische Unterschiede beschrieben und diskutiert.

Empirische Studien belegen, dass Männer kulturübergreifend häufiger körperliche Aggression zeigen als Frauen [22, 30]. Diese Tendenz spiegelt sich ebenfalls in den aktuellen Daten der deutschen Bundeskriminalstatistik 2024 wider, wo Männer bei Gewaltstraftaten deutlich häufiger als Tatverdächtige erfasst werden als Frauen [6]. Im Jahr 2024 wurden bei Körperverletzungen 3,5-mal so viele Männer wie Frauen und bei schwerer und gefährlicher Körperverletzung 4,5-mal so viele als Tatverdächtige registriert.

Zur Erklärung dieser Unterschiede werden verschiedene theoretische Ansätze herangezogen. Eagly und Steffen [12] führen geschlechtsspezifische Unterschiede bei körperlicher Aggression primär auf soziale Rollenverteilungen und erlernte Normen zurück. Frauen werden in vielen Kulturen schon früh zu prosozialem und konfliktvermeidendem Verhalten erzogen. Denson et al. [11] argumentieren, dass Frauen die potenziellen negativen Konsequenzen körperlicher Aggression für sich selbst oder andere stärker antizipieren, was angesichts häufig unterschiedlicher körperlicher Kraftverhältnisse eine hemmende Wirkung auf aggressives Verhalten haben kann.

Neben der körperlichen Aggression, die sich meist durch direktes und sichtbares Verhalten äußert, existieren auch indirekte Formen aggressiven Verhaltens, die subtiler und verdeckter auftreten (z. B. Mobbing oder soziale Ausgrenzung). Studien legen nahe, dass Frauen im Vergleich zu Männern häufiger zu indirekten Aggressionsformen neigen [11, 21]. Insbesondere in der Kindheit und Jugend ist dieser Geschlechterunterschied ausgeprägt [21, 38]. Im Erwachsenenalter scheinen diese Geschlechtsunterschiede jedoch nicht mehr zu bestehen [2], was unter anderem an veränderten sozialen Rollen und Normen liegen könnte.

## Einflussfaktoren

Unabhängig von den unterschiedlichen Erscheinungsformen wird aggressives Verhalten multifaktoriell unter anderem durch biologische (genetische Disposition, Epigenetik), kulturelle, situative, lernpsychologische (z. B. Bildung) und personenspezifische (Trait‑)Variablen beeinflusst.

Die Bedeutung situativer Faktoren wird exemplarisch in Laborstudien deutlich. Einige Untersuchungen belegen, dass Männer unter neutralen Bedingungen tendenziell aggressiver reagieren als Frauen. Dieser Unterschied verringert sich jedoch mit zunehmender Provokation im kontrollierten Laborsetting oder löst sich vollständig auf [11, 44]. Diese Ergebnisse deuten darauf hin, dass Frauen unter bestimmten situativen Bedingungen ähnliche Reaktionsmuster zeigen können. Bislang sind diese Befunde jedoch auf Studien in einem kontrollierten experimentellen Kontext zur Erfassung reaktiver Aggression beschränkt, einer Form, die die Reaktion auf Provokation, Frustration und Bedrohung charakterisiert. Darüber hinaus scheinen Männer aggressives Verhalten häufiger zu initiieren, was teilweise auf eine stärkere Impulsivität zurückgeführt werden kann; eine experimentelle Verzögerung der Reaktion reduziert aggressive Antworten deutlich [27].

Auch (neuro-)biologische Einflussfaktoren tragen zur Erklärung aggressiven Verhaltens bei. Bei Männern zeigt sich ein schwacher, aber positiver Zusammenhang zwischen dem Baseline-Testosteronspiegel und aggressivem Verhalten – ebenso bei situativ bedingten Veränderungen des Testosteronspiegels [16]. So hängt Aggression stärker von kontextabhängigen Schwankungen des Testosteronspiegels infolge von Wettbewerb oder sozialer Provokation ab als von den zirkadianen Testosteronschwankungen [7]. Ferner deuten neurobiologische Studien auf geschlechtsspezifische Aktivitätsmuster hin: Unter hoher Provokation weisen Männer eine verstärkte Aktivierung der Amygdala auf, die mit höheren Trait-Ärger-Werten korreliert ist [36].

Insgesamt kann Aggression im Sinne eines biopsychosozialen Modells wie dem General Aggression Model [1] als Resultat wechselseitiger Einflüsse biologischer, psychologischer, Umwelt- und sozialer Faktoren verstanden werden. Geschlecht wirkt hierbei nicht als monofaktorieller Einfluss, sondern interagiert mit biologischen Dispositionen (z. B. hormonelle Einflüsse wie Testosteron), psychologischen Prozessen (z. B. Impulskontrolle, Emotionsregulation) sowie Umwelt- (frühkindliche Missbrauchserfahrungen) und sozialen Faktoren (z. B. Geschlechtserwartungen und soziale Normen). Diese Einflussfaktoren prägen Aggression nicht nur bei gesunden Personen, sondern werden im Kontext psychischer Störungen relevant, da Aggression hier als relevantes klinisches Merkmal auftreten kann.

Psychische Störungen könnten auf den biopsychosozialen Ebenen wirken (z. B. hormonell/neurobiologisch durch Beeinträchtigung kognitiver und emotionaler Regulation oder durch Veränderungen sozialer Interaktionen) und dadurch bestehende Geschlechtsunterschiede in aggressivem Verhalten modifizieren. Wie genau diese Mechanismen funktionieren und in welchen Störungen spezifische Muster auftreten, ist bislang nur unzureichend erforscht und bedarf systematischer Untersuchungen.

## Geschlechtsunterschiede in aggressivem Verhalten bei psychischen Störungen

Groß angelegte Reviews und Registerstudien belegen, dass Aggression bei psychischen Störungen als transdiagnostisches Phänomen symptomatisch auftreten kann. Dazu zählen insbesondere die Borderline- (BPS; [24]) und antisoziale Persönlichkeitsstörung [17], Aufmerksamkeitsdefizit‑/Hyperaktivitätsstörung (ADHS; [40]), Autismusspektrumstörung [14], psychotische Störungen [23], posttraumatische Belastungsstörung (PTBS; [33]) sowie Substanzabhängigkeitsstörungen [39].

Daten aus populationsbasierten Studien zeigen, dass Personen mit psychischen Störungen ein ca. 3,5-mal höheres Risiko haben, Opfer oder Täter von Gewalt zu werden, verglichen mit ihren psychisch gesunden Geschwistern [39]. Eigene Viktimisierungserfahrungen können ebenfalls das Risiko für aggressives Verhalten erhöhen [37]. Dieses Phänomen wird in der Literatur als „victim-offender overlap“ bezeichnet [19] und beschreibt, dass Personen, die Gewalt ausüben, häufiger auch selbst Viktimisierung erfahren haben.

Zudem zeigt sich geschlechtsspezifisch, dass Männer mit psychischen Störungen insgesamt ein höheres absolutes Risiko sowohl für Viktimisierung als auch für Täterschaft aufweisen als Frauen [39]. Außerdem sind Frauen mit psychischen Störungen häufiger Opfer als Täterinnen, während Männer mit psychischen Störungen häufiger Täter als Opfer sind [9].

In den folgenden Abschnitten werden exemplarisch drei psychische Störungsbildern mit hoher klinischer Relevanz – Substanzabhängigkeitsstörungen, psychotische Störungen und BPS – empirisch-deskriptiv dargestellt.

### Substanzabhängigkeitsstörungen

#### Klinischer Steckbrief.

- Kerndiagnosekriterien: Kontrollverlust über den Konsum, risikoreicher Konsum, soziale Beeinträchtigung [47]
- Männer sind mehr als doppelt so häufig betroffen wie Frauen [10].

Aggressives Verhalten wird im Kontext von Substanzabhängigkeitsstörungen häufig als Begleitsymptom beschrieben. Personen mit Substanzabhängigkeitsstörungen fallen im Vergleich zur Allgemeinbevölkerung durch erhöhte Werte bei verschiedenen Formen aggressiven Verhaltens auf [3], darunter körperliche und verbale Aggression sowie Feindseligkeit und Ärger.

Eine differenzierte Betrachtung geschlechtsspezifischer Muster bietet die Studie von Bácskai et al. [3]. Frauen mit einer Marihuanaabhängigkeit schätzen ihre Feindseligkeit und ihren Ärger höher ein als Männer. Bei verbaler und körperlicher Aggression hingegen zeigen sich keine signifikanten Unterschiede. Dieses Muster gilt jedoch ausschließlich für Marihuana; bei Alkohol- und Heroinabhängigkeit sind keine bedeutsamen Unterschiede zwischen den Geschlechtern festzustellen. Interessanterweise korreliert chronischer Substanzkonsum bei Frauen mit einem höheren Maß an Aggression im Vergleich zur Allgemeinpopulation. Die Autoren diskutieren, ob chronischer Substanzkonsum bei Frauen eher aggressives Verhalten provoziert als bei Männern bzw. die Hemmung zu aggressivem Verhalten durch den Substanzkonsum aufgehoben wird.

Die Wahrscheinlichkeit gewalttätigen Verhaltens ist bei Männern mit Substanzabhängigkeitsstörungen sowohl in Konflikten in ihrer Partnerschaft als auch in Auseinandersetzungen mit anderen Personen (z. B. Freunden, Fremden) höher [8].

Kraanen et al. [20] zeigen ergänzend, dass verschiedene Kombinationen von Substanzen (sog. Polysubstanzkonsum) das Risiko für Partnerschaftsgewalt auf unterschiedliche Weise bei Männern und Frauen beeinflussen. Männliche Patienten, die alkoholabhängig sind und zusätzlich Cannabis, Kokain oder beide Substanzen konsumieren, haben gegenüber ausschließlich alkoholabhängigen Männern ein erhöhtes Risiko für *jegliche sowie schwere Partnerschaftsgewalt*. Bei Patientinnen ist die gleichzeitige Abhängigkeit von Alkohol und Kokain mit einem erhöhten Risiko für *jegliche Partnerschaftsgewalt* verbunden, während Alkohol in Kombination mit Cannabis das Risiko für *schwere Partnerschaftsgewalt* erhöht.

Ergänzend zu diesen klinischen Studien liefern auch forensische Daten Hinweise auf geschlechtsspezifische Unterschiede: So konnten de Vogel et al. [43] in einer Studie mit forensischen Patientinnen und Patienten feststellen, dass Männer zum Zeitpunkt der Straftat, die zur Einweisung in die Forensik führte, signifikant häufiger unter dem Einfluss von Alkohol oder Drogen standen als Frauen, was auch dem allgemeinen geschlechtsspezifischen Konsummuster mit häufigerem Alkoholkonsum bei Männern entspricht [45].

### Psychotische Störungen

#### Klinischer Steckbrief.

- Kerndiagnosekriterien: Wahn, Halluzinationen, desorganisiertes Denken [47]
- Bei Männern deutlich häufiger als bei Frauen, insbesondere im jungen Erwachsenenalter mehr als doppelt so oft [31]

Aggressives Verhalten, insbesondere körperliche Aggression, tritt auch und vor allem bei Schizophrenie und anderen psychotischen Störungen vermehrt auf. Eine Metaanalyse von Fazel et al. [13] findet ein Chancenverhältnis (Odds Ratio [OR]) von 3,98 für gewalttätiges Verhalten bei Männern und 7,85 bei Frauen mit Schizophrenie im Vergleich zu gesunden Kontrollpersonen (vgl. auch Yee et al. [46] für eine aktuellere Metaanalyse mit ähnlichem Muster). Auch umfangreiche Register- und populationsbasierte Studien, wie die von Fleischman et al. [15], unterstreichen die erhöhten Risiken für beide Geschlechter. Dabei offenbart sich ein wiederkehrendes Muster: Frauen mit psychotischen Störungen weisen ein mindestens vergleichbares, in einigen Fällen sogar höheres Gewaltrisiko auf, das in den Studien als Gewaltstraftaten wie Mord, Totschlag, Raub oder Belästigung erfasst wird. Es ist jedoch wichtig zu beachten, dass Männer in der Allgemeinbevölkerung insgesamt häufiger gewalttätig sind, sodass bei Frauen bereits ein geringerer absoluter Anstieg zu einem erhöhten Chancenverhältnis führt.

Darüber hinaus verdeutlichen die Ergebnisse von Fazel et al. [13] auch die Relevanz komorbider Substanzabhängigkeit. Die Metaanalyse zeigt eine deutlich erhöhte Wahrscheinlichkeit für gewalttätiges Verhalten bei Patientinnen und Patienten mit komorbider Substanzabhängigkeit. Interessanterweise ist das Risiko bei Personen mit alleiniger Substanzabhängigkeit nicht geringer als bei jenen mit komorbider psychotischer Störung. Dies weist darauf hin, dass eine Abhängigkeitserkrankung einen entscheidenden Erklärungswert für aggressives Verhalten besitzt. Die Autoren betonen jedoch, dass dieser Zusammenhang auch durch weitere Einflussfaktoren wie Persönlichkeitsmerkmale oder soziale Belastungsfaktoren vermittelt sein könnte.

Ein Geschlechtervergleich stationär behandelter Patientinnen und Patienten mit psychotischen Störungen, die unter Einsatz von Zwangsmaßnahmen behandelt wurden, ergab keine Unterschiede hinsichtlich der fremdbeurteilten Aggressivität [28]. Eine Subgruppenanalyse zeigt jedoch, dass Männer mit besonders hoher fremdbeurteilter Aggressivität innerhalb dieser Subgruppe signifikant höhere Werte als Frauen aufweisen, was auf geschlechtsspezifische Unterschiede in der Intensität aggressiven Verhaltens hinweist.

Ergänzend stellen Steinert et al. [42] fest, dass männliche Patienten während ihrer ersten Hospitalisierung sowohl körperlich als auch verbal aggressiver auftreten als weibliche Patientinnen. Dieser Geschlechtsunterschied tritt jedoch bei späteren Klinikaufenthalten nicht mehr auf.

### Borderline-Persönlichkeitsstörung

#### Klinischer Steckbrief.

- Kerndiagnosekriterien: Emotionale Instabilität, Impulsivität, instabile Beziehungen, häufige Ärgergefühle, suizidale Tendenzen und Selbstverletzungen, überdauernd seit der Jugend [47]
- In der Allgemeinbevölkerung sind Geschlechtsunterschiede bei BPS bisher unklar, in klinischen Stichproben liegt das Verhältnis häufig bei etwa 3:1 (Frauen:Männer; [5]).

Eine gestörte Ärgerregulation und damit verbundene Verhaltensweisen wie körperliche Auseinandersetzungen zählen zu den zentralen Merkmalen der BPS [47]. In Studien berichten 73 % der Betroffenen innerhalb eines Jahres von aggressivem Verhalten, 58 %, gelegentlich oder häufig in körperliche Auseinandersetzungen verwickelt gewesen zu sein, und 25 %, bereits eine Waffe gegen andere eingesetzt zu haben [29]. Mit einer geschätzten Prävalenz von 30 % stellen Personen mit BPS einen erheblichen Anteil der Gefängnispopulation dar [4].

Die erhöhte reaktive Aggressivität bei BPS kann sowohl durch kategoriale [26] als auch durch dimensionale Messungen [32] der Symptomatik nachgewiesen werden und gilt daher als zentrales Merkmal dieser Störung. Vier Faktoren sind hier von Bedeutung: gestörte Ärgerregulation als Facette der Affektregulationsstörung, Impulsivität, soziale Bedrohungshypersensitivität, reduzierte kognitive Empathie sowie auch Ansteckungsphänomene im Kontext von Mehrpersonensettings [25].

Ähnlich wie bei anderen psychischen Störungen deutet sich auch bei der BPS ein geringerer Geschlechtsunterschied gegenüber gesunden Stichproben an [24, 34]. Lediglich im Bereich körperlicher Aggression können höhere Werte bei männlichen gegenüber weiblichen Betroffenen gefunden werden, während kein Unterschied bei verbaler Aggression, Wut und Feindseligkeit berichtet wird [41]. Darüber hinaus liegen Hinweise auf häufigeres externalisierendes Verhalten bei männlichen gegenüber weiblichen Betroffenen vor [34]. Erhöhte Testosteronwerte in Ruhe werden sowohl bei männlichen als auch weiblichen Personen mit BPS berichtet, sie stehen jedoch nicht im Zusammenhang mit selbstberichteter Aggressivität [35]. Die Ergebnisse aus dieser Studie weisen jedoch auf erhöhte Kortisolaufwachreaktionen bei weiblichen, aber nicht bei männlichen Patienten mit BPS hin im Vergleich zu Gesunden. Bei den Frauen waren dabei die Kortisolaufwachreaktionen positiv mit Wut und Aggressivität assoziiert.

Ein Beispiel aus einer neurobiologischen Studie zeigt teilweise geschlechtsspezifische Unterschiede. Während eines Imaginationsexperiments in einem Magnetresonanztomographen, bei dem die Patientinnen und Patienten sich vorstellen sollten, nach einer Zurückweisung aggressiv zu reagieren, weisen Männer mit BPS stärkere Aktivierungen in der Amygdala und in den präfrontalen Arealen auf als Frauen mit BPS und gesunde Männer [18]. Trait-Ärger moduliert bei männlichen Patienten die Koppelung zwischen präfrontalen Regionen und der Amygdala negativ, was auf eine schlechte Top-down-Anpassung des Verhaltens bei männlichen Patienten mit BPS hinweisen könnte: Das Ärgererleben bleibt trotz der Bemühungen um Kontrolle präsent. Demgegenüber fällt die präfontoamygdalare Kopplung bei den weiblichen Patientinnen positiv aus. Sie konnten in diesem Experiment (Zurückweisung durch einen Peer) die aggressive Spannung während der Imagination aggressiven Verhaltens erfolgreich dämpfen.

## Schlussfolgerung

Die narrative Literaturübersicht zu Geschlechtsunterschieden im aggressiven Verhalten bei ausgewählten psychischen Störungen, darunter Substanzabhängigkeitsstörungen, psychotische Störungen und BPS, zeichnet ein differenziertes Bild. Bei Substanzabhängigkeitsstörungen ist die Prävalenz aggressiven Verhaltens insgesamt hoch. Geschlechtsspezifische Unterschiede werden dabei unter anderem durch multiplen Substanzkonsum und konflikthafte Beziehungskonstellationen beeinflusst. Frauen mit psychotischen Störungen weisen ein erhöhtes Risiko für gewalttätiges Verhalten auf, das mindestens dem von Männern mit psychotischen Störungen entspricht und teilweise sogar höher liegt. Bei der BPS zeigt sich eine höhere Prävalenz körperlicher Aggression bei Männern.

Insgesamt erweist sich das Konstrukt Aggression als ein komplexes, multifaktoriell beeinflusstes Phänomen, das transdiagnostisch relevant ist. In der Forschung erlaubt ein biopsychosoziales Modell wie das General Aggression Model die Berücksichtigung biologischer, psychologischer und sozialer Faktoren sowie deren Wechselwirkungen. Dabei kann das Geschlecht nicht isoliert, sondern muss im Zusammenspiel mit biopsychosozialen Faktoren betrachtet werden. Psychische Störungen könnten diese Faktoren zusätzlich verändern, indem sie hormonelle Prozesse modulieren, die Regulationsfähigkeit beeinträchtigen und soziale Interaktionen verändern. Dies könnte eine mögliche Erklärung liefern, warum sich Geschlechtsunterschiede bei Aggression von denen der Allgemeinbevölkerung unterscheiden. Wie genau diese Prozesse in den Störungsbildern ablaufen, und auch wie sich dies über die Lebensspanne verändert, ist bislang unzureichend erforscht.

Die Mehrheit der empirischen Befunde basiert bislang auf Selbst- und Fremdberichten, während verhaltensbasierte Erhebungsansätze kaum verfügbar sind und in zukünftigen Studien verstärkt berücksichtigt werden sollten. Auch bieten Registerstudien grundsätzlich Potenzial für weiterführende Analysen, konzentrieren sich jedoch bislang überwiegend auf Unterschiede zwischen Personen mit und ohne spezifische psychische Störung, während Geschlechtsunterschiede hierbei noch kaum untersucht wurden.

Ein besseres Verständnis geschlechtsspezifischer Unterschiede wäre auch klinisch relevant, sowohl für die Diagnostik als auch für die Prädiktion und die individualisierte Therapieplanung. Gegenwärtig kann eine differenzierte Erfassung aggressiven Verhaltens durch Situationsanalysen (z. B. nach dem SORKC-Modell) erfolgen, um Aggression im Gesamtkontext der Störung zu verstehen und therapeutisch zu adressieren.

Die vorliegende Arbeit ist als narrative Literaturübersicht zu verstehen, die die bestehende teilweise knappe relevante Literatur wiedergibt. Zudem konnten nicht alle relevanten Störungsbilder, bei denen aggressives Verhalten eine Rolle spielt, berücksichtigt werden. Insbesondere die antisoziale Persönlichkeitsstörung, die für aggressives Verhalten von besonderer Bedeutung ist, wurde im vorliegenden Beitrag nicht behandelt. Eine vertiefte Auseinandersetzung mit anderen klinisch relevanten Diagnosen sollte in zukünftigen, idealerweise systematischen Übersichtsarbeiten erfolgen.

## Fazit für die Praxis

- Aggression ist ein komplexes, multifaktoriell beeinflusstes Phänomen, das transdiagnostisch relevant ist.
- Ein biopsychosoziales Modell wie das General Aggression Model erlaubt die Berücksichtigung biologischer, psychologischer und sozialer Faktoren sowie deren Wechselwirkungen. Das Geschlecht kann nicht isoliert, sondern muss im Zusammenspiel mit biopsychosozialen Faktoren betrachtet werden.
- Psychische Störungen könnten diese Faktoren zusätzlich verändern, indem sie hormonelle Prozesse modulieren, die Regulationsfähigkeit beeinträchtigen und soziale Interaktionen verändern.
- Praktisch kann eine differenzierte Erfassung aggressiven Verhaltens durch Situationsanalysen (z. B. nach dem SORKC-Modell) erfolgen, um Aggression im Gesamtkontext der Störung zu verstehen und therapeutisch zu adressieren.
